# Enabling Chemical Holography: A Comprehensive Framework and Implementation

**DOI:** 10.1109/jphot.2025.3567016

**Published:** 2025-05-05

**Authors:** Shihao Ran, David Mayerich, Rohith Reddy

**Affiliations:** The authors are with the Department of Electrical and Computer Engineering, University of Houston, Houston, TX 77204 USA

**Keywords:** Holography, mid-infrared, mid-infrared spectroscopic imaging, MIRSI, phase imaging, phase microscopy, phase sensitive, spectroscopy

## Abstract

Mid-infrared spectroscopic imaging (MIRSI) enables the spatially-resolved identification of molecules and is widely used in fields ranging from biomedical diagnostics to forensics. Current MIRSI technologies measure the sample’s extinction coefficient, which is only one component of the complex relative permittivity, and therefore provide incomplete molecular profiles. We propose a new framework and instrument to enable phase-sensitive *chemical holography* that measures a sample’s *complex* molecular properties at any wavelength, thus overcoming a fundamental limit on molecular specificity. Combining a spatially coherent quantum cascade laser (QCL) source with an interferometer and imaging system can provide a phase-sensitive platform for molecular analysis. This paper describes a theoretical framework for chemical holography and demonstrates benefits for molecular specificity, improved spatial resolution, and greater flexibility. Deep learning is used to solve the inverse scattering problem for chemically heterogeneous samples modeled using Mie theory. Furthermore, we demonstrate new, custom-built instrumentation and experimental results that validate our theoretical framework.

## I. Introduction

MID-INFRARED (mid-IR) spectroscopy is a nondestructive label-free method for quantitatively measuring molecular bonds [1]. The resulting absorbance spectrum provides a signature used to identify chemical components in homogeneous samples. Mid-IR spectroscopic imaging (MIRSI) extends this technique to provide spatial resolution in heterogeneous samples [1], [2], [3], [4], [5], [6], [7], [8]. Recent research demonstrates the viability of infrared microscopy for characterizing complex biomedical samples at cellular-level resolution [9], [10], [11], [12], [13], [14], [15], [16], [17].

However, infrared spectra are often distorted in heterogeneous samples due to scattering, which poses a challenge for molecular identification. A variety of *ad hoc* [18] and physically-based [19] methods attempt to compensate for these artifacts. However, their success is limited because mid-infrared absorbance only measures the imaginary component of the refractive index. This incomplete information severely limits the ability to build inverse models, which would provide more specific molecular information.

This paper provides a theoretical framework for *chemical holography* to enable spatially and spectrally resolved phase-sensitive measurements of heterogeneous samples that facilitates measurement of the sample’s complex refractive index. We also demonstrate novel, custom-built instrumentation based on the proposed framework and present experimental results supporting the application of chemical holography. Our aim is to provide a theoretical foundation that can be extended to areas such as infrared spectroscopy and label-free molecular identification.

## II. Background

IR spectroscopy produces a hyperspectral absorbance image as a function of wavenumber $\overset{‾}{\nu }$ (in ${\text{cm}}^{-1}):\;A(x,y,\overset{‾}{\nu })=-{\text{log}}_{10}\frac{I(x,y,\overset{‾}{\nu })}{I_{0}(x,y,\overset{‾}{\nu })}$, where $I_{0}(x,y,\overset{‾}{\nu })$ is a hyperspectral image of the source and $I(x,y,\overset{‾}{\nu })$ is an image of the sample. The resulting absorbance image A is proportional to the spatially-resolved extinction coefficient $\kappa (x,y,\overset{‾}{\nu })$ of the wavenumber-dependent complex refractive index:

(1) $$n(x,y,\overset{‾}{\nu })=m(x,y,\overset{‾}{\nu })+i\kappa (x,y,\overset{‾}{\nu })$$

Since most samples are non-magnetic, the refractive index is directly related to the material’s electric susceptibility: ${𝒳}_{e}={𝒳}_{e}^{\prime }+i{𝒳}_{e}^{\prime \prime }=n^{2}-1$. Absorbance measurements only provide an approximation of κ, fundamentally limiting the estimation of ${𝒳}_{e}$. This information is further distorted by scattering induced through spatial inter-dependencies within a heterogeneous sample: $n(x,y,\overset{‾}{\nu })$.

## III. Forward Model

We first describe the forward models that form the foundation for chemical holography, which are based on coupled wave [20], [21], [22], [23], [24] and Mie theory [25], [26]. The incident light is modeled as a coherent infrared source, such as a quantum cascade laser (QCL) with an intensity spectrum given by $I_{0}(\overset{‾}{\nu })$. Using Maxwell’s equations [27], the electric field E in a vacuum $\nabla^{2}E-\mu_{0}\epsilon_{0}\frac{\partial^{2}E}{\partial t^{2}}=0$ has a solution: $E(r,t)=E_{0}\text{cos}(k\cdot r-\omega t+\phi )$. where $E_{0}$ is the complex amplitude and k is the propagation vector such that $|k|=2\pi \overset{‾}{\nu }$. This is generally simplified using the initial phase ϕ=0 and time constant $t=0:E(r)=E_{0}e^{ik\cdot r}$. Incorporating the complex refractive index (1) into the electric field equation provides an expression for its propagation within a medium: $E(r,\overset{‾}{\nu })=E_{0}e^{ik\cdot rn(\overset{‾}{\nu })}$. Note that the refractive index n is a physical response function, therefore the bi-directional Kramers-Kronig relation links the real and imaginary parts [28]. Specifically, for a complex function $\chi (\omega )=\chi_{1}(\omega )+i\chi_{2}(\omega )$, the real and imaginary components are related by:

(2) $$\chi_{1}(\omega )=\frac{1}{\pi }P\int_{-\infty }^{\infty }\frac{\chi_{2}\left(\omega^{\prime }\right)}{\omega^{\prime }-\omega }d\omega^{\prime }$$

(3) $$\chi_{2}(\omega )=-\frac{1}{\pi }P\int_{-\infty }^{\infty }\frac{\chi_{1}\left(\omega^{\prime }\right)}{\omega^{\prime }-\omega }d\omega^{\prime }$$

and therefore provide an additional set of constraints. Finally, the electric field is expressed as independent components $E={\left[E_{x},E_{y},E_{z}\right]}^{T}\in C^{3}$ with the measured intensity $I=E^{H}E$, where H signifies the Hermetian transpose.

### A. Layered Samples

We use a coupled-wave approach to simulate a layered homogeneous sample [20]. The electric and magnetic fields are given by $E(r,\overset{‾}{\nu })=E_{0}e^{ik\cdot rn(\overset{‾}{\nu })}$ and $B=\sqrt{\frac{\epsilon_{0}}{\mu_{0}}}\left(s\times E_{0}\right)e^{\text{ik}s\cdot r}$, where s is the normalized propagation direction vector scaled by the complex refractive index in layer-$\ell :s_{x}^{2}+s_{y}^{2}+s_{z}^{2}=\epsilon (\lambda )=n(\overset{‾}{\nu }{)}^{2}$, and the $s_{z}$ component for any layer ℓ is given by the dispersion relation: $s_{z}^{\left(\ell \right)}=\sqrt{n_{\ell }^{2}(\overset{‾}{\nu })-s_{x}^{2}-s_{y}^{2}}$. The boundaries of each layer are defined by z-coordinates such that layer ℓ∈[0,L] runs from $z_{\ell }$ to $z_{\ell +1}$. The first (ℓ=0) and last (ℓ=L) layers are unbounded so that $z_{L+1}$ is undefined and $z_{0}$ is the position of a specified input field. The field at any point within layer ℓ is the sum of ${\overset{ˇ}{P}}^{\left(\ell \right)}$ propagating along -z from $z_{\ell +1}$ and ${\overset{ˆ}{P}}^{\left(\ell \right)}$ propagating along +z from $z_{\ell }$:

(4) $$E^{\left(\ell \right)}(z)\;=\left[{\overset{ˇ}{P}}^{\left(\ell \right)}e^{\text{ik}s_{z}^{\left(\ell \right)}\left(z-z^{\ell }\right)}+{\overset{ˆ}{P}}^{\left(\ell \right)}e^{-\text{ik}s_{z}^{\left(\ell \right)}\left(z-z^{\ell +1}\right)}\right]e^{\text{ik}\left(s_{x}x+s_{y}y\right)}$$

Enforcing Gauss’ Law across boundaries gives:

(5) $$s_{x}{\overset{ˇ}{P}}_{x}^{\left(\ell \right)}+s_{y}{\overset{ˇ}{P}}_{y}^{\left(\ell \right)}+s_{z}^{\left(\ell \right)}{\overset{ˇ}{P}}_{z}^{\left(\ell \right)}=0$$

(6) $$s_{x}{\overset{ˆ}{P}}_{x}^{\left(\ell \right)}+s_{y}{\overset{ˆ}{P}}_{y}^{\left(\ell \right)}+s_{z}^{\left(\ell \right)}{\overset{ˆ}{P}}_{z}^{\left(\ell \right)}=0$$

The field E is continuous across layer boundaries, and the following two exceptions apply:

1. The initial input field ${\overset{ˇ}{P}}^{\left(0\right)}$ is specified at $z_{0}$ inside ℓ=0.
2. The final boundary is the interface between layers (L-2) and (L-1), therefore there is no negative-propagating field in (L-1).

This results in L layers defined by L-1 boundaries with the input given at $z_{0}$. The following equations enforce continuity across these boundaries:

(7) $$A{\overset{ˇ}{P}}_{x}^{\left(\ell -1\right)}+{\overset{ˆ}{P}}_{x}^{\left(\ell \right)}-{\overset{ˇ}{P}}_{x}^{\left(\ell \right)}-B{\overset{ˆ}{P}}_{x}^{\left(\ell \right)}=0$$

(8) $$A{\overset{ˇ}{P}}_{y}^{\left(\ell -1\right)}+{\overset{ˆ}{P}}_{y}^{\left(\ell \right)}-{\overset{ˇ}{P}}_{y}^{\left(\ell \right)}-B{\overset{ˆ}{P}}_{y}^{\left(\ell \right)}=0$$

(9) $$As_{y}{\overset{ˇ}{P}}_{z}^{\left(\ell -1\right)}-As_{z}^{\left(\ell -1\right)}{\overset{ˇ}{P}}_{y}^{\left(\ell -1\right)}+s_{y}{\overset{ˆ}{P}}_{z}^{\left(\ell -1\right)}+s_{z}^{\left(\ell -1\right)}{\overset{ˆ}{P}}_{y}^{\left(\ell -1\right)}\;=s_{y}{\overset{ˇ}{P}}_{z}^{\left(\ell \right)}-s_{z}^{\left(\ell \right)}{\overset{ˇ}{P}}_{y}^{\left(\ell \right)}+Bs_{y}{\overset{ˆ}{P}}_{z}^{\left(\ell \right)}+Bs_{z}^{\left(\ell \right)}{\overset{ˆ}{P}}_{y}^{\left(\ell \right)}$$

(10) $$As_{z}^{\left(\ell -1\right)}{\overset{ˇ}{P}}_{z}^{\left(\ell -1\right)}-As_{x}{\overset{ˇ}{P}}_{z}^{\left(\ell -1\right)}-s_{z}^{\left(\ell -1\right)}{\overset{ˆ}{P}}_{x}^{\left(\ell -1\right)}-s_{x}{\overset{ˆ}{P}}_{z}^{\left(\ell -1\right)}\;=s_{z}^{\left(\ell \right)}{\overset{ˇ}{P}}_{x}^{\left(\ell \right)}-s_{x}{\overset{ˇ}{P}}_{z}^{\left(\ell \right)}-Bs_{z}^{\left(\ell \right)}{\overset{ˆ}{P}}_{x}^{\left(\ell \right)}-Bs_{x}{\overset{ˆ}{P}}_{z}^{\left(\ell \right)}$$

where

(11) $$A=e^{\text{ik}s_{z}^{\left(\ell -1\right)}\left(z_{l}-z_{\ell -1}\right)}$$

(12) $$B=e^{-\text{ik}s_{z}^{\left(\ell \right)}\left(z_{l}-z_{\ell +1}\right)}$$

Eqs. (5)–(10) form a linear system with 6(L-1) independent equations and unknowns.

### B. Mie Scattering

**Fig. 1.**
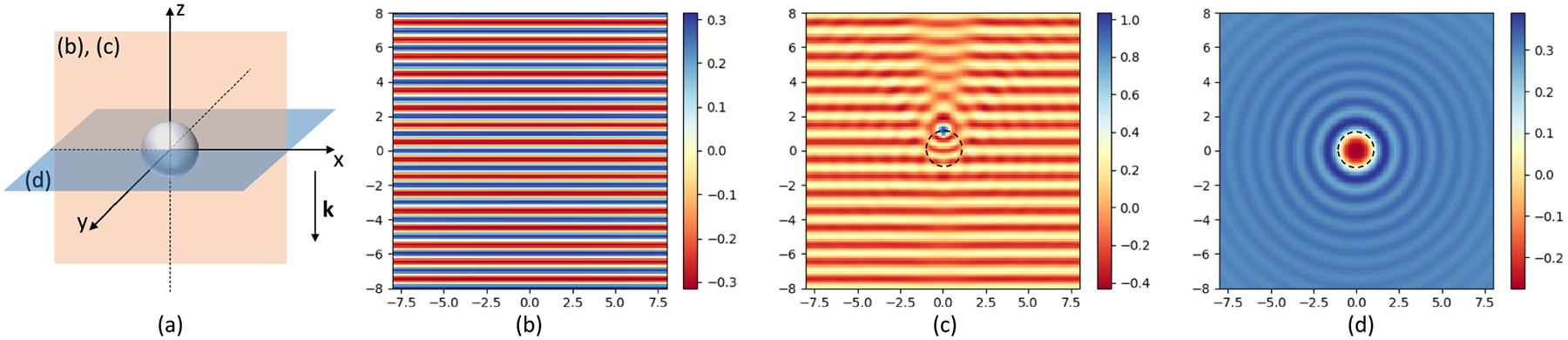
Traditional Mie scattering simulation for a sphere with radius a=1μm, complex refractive index n=1.5+i0.03. (a) The coordinate system of the simulation, the incident plane wave is propagating along -z axis denoted by the black arrow k. The sphere is positioned at the origin. The orange plane is the visualization plane for figures (b) and (c), and the blue plane for figure (d). (b) The incident field. (c) The vertical view of the total field. (d) The horizontal view. The outline of the sphere is denoted by the black dashed line. The wavelength of the incident plane wave λ=1μm, and the FOV is 16μm×16μm. Only the real part of the field is shown in arbitrary units [a.u.].

Mie theory [29] was selected to model heterogeneous samples due to its wide applicability in IR imaging to account for cellular-level scattering [19], [30]. The field produced by a sphere is defined as the summation of incident $\left(E_{I}\right)$ and scattered $\left(E_{S}\right)$ components (Fig. 1): $E(r,\overset{‾}{\nu })=E_{I}(r,\overset{‾}{\nu })+E_{S}(r,\overset{‾}{\nu })$. The input field $E_{I}$ is provided as input to the model and the scattered field is computed as:

(13) $$E_{S}(p)=\sum_{\ell =0}^{\infty }B_{\ell }(\overset{‾}{\nu },n,a)h_{\ell }^{\left(1\right)}(\text{kr})P_{\ell }(\text{cos}\theta )$$

where the scalar value B enforces continuity across the sphere boundary with respect to the internal field [31]:

(14) $$B\left(\bar{v},n,a\right)=\left(2t+1\right)i^{t}\times \frac{j_{t}\left(\text{ka}\right)j_{t}^{\prime }\left(\text{kna}\right)n-j_{t}\left(\text{kna}\right)j_{t}^{\prime }\left(k\right)}{j_{t}\left(\text{kna}\right)h_{t}^{\left(1\right)}\left(\text{ka}\right)-h_{t}^{{\left(1\right)}^{\prime }}\left(\text{ka}\right)-h_{t}^{\left(1\right)}\left(\text{ka}\right)j_{t}^{\prime }\left(\text{kna}\right)n}$$

where $P_{t}$ is the order-t Legendre polynomial, $j_{t}$ is the spherical Bessel function, and $h_{t}^{\left(1\right)}$ denotes the spherical Hankel function of the first kind [32]. The corresponding asymptotic expressions [30] give the far-field when z≫a:

(15) $$E_{F}(p)=\sum_{t=0}^{\infty }B_{t}(\lambda ,n,a)\frac{e^{\left[i(\text{kr}-t\pi /2)\right]}}{i\text{kr}}P_{t}\left(\sqrt{1-k_{\|}^{2}}\right)$$

**Fig. 2.**
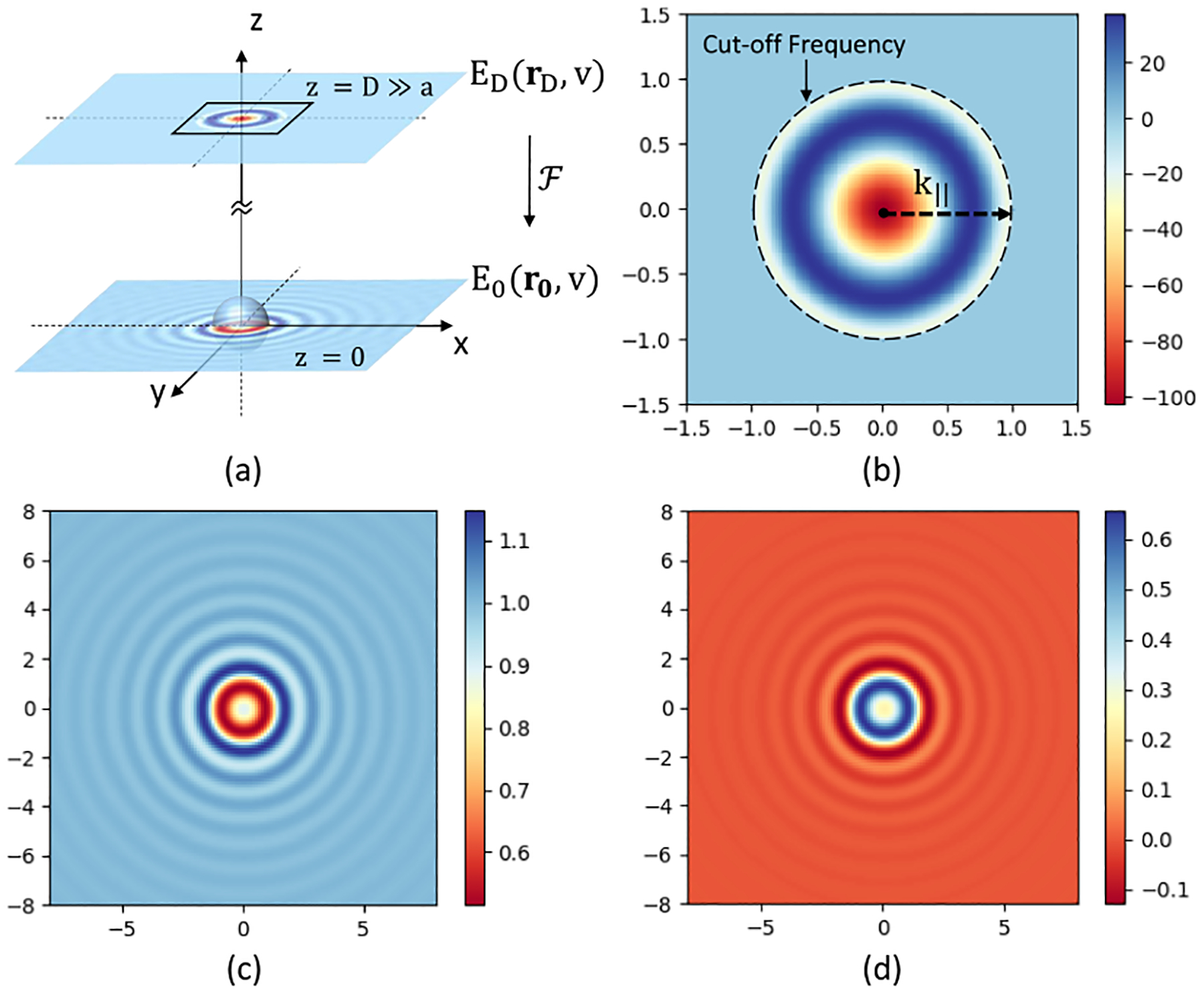
Far-field simulation of the same sphere specified in Fig. 1. (a) Calculation diagram, (b) the real component of the far field (zoom-in), (c) the real component of the near field, (d) the imaginary component of the near field I scattered by the sphere. The FOV of the images is 16μm×16μm.

This result is then band-limited by the objective aperture (NA) and the resulting far-field image is calculated using the Fourier transform $E_{F}\left(r_{F},\overset{‾}{\nu }\right)\overset{ℱ}{\to }E(r,\overset{‾}{\nu })$ (Fig. 2). In simulations, the critical characteristic is the ratio of the sample feature size (ex. bead diameter) to wavelength. Since infrared light is frequently used to probe structures with sizes in the micrometer range, we present simulations at a wavelength of λ=1μm to provide the reader with a convenient “yardstick”. The mathematics are identical for any infrared wavelength as long as the appropriate material parameters (ex. refractive index) are known.

### C. Relating Optical Measurements to Material Properties

The measured absorbance of a homogeneous sample obeys the Beer-Lambert law [33]:

(16) $$A(\overset{‾}{\nu })=\rho \times b\times \epsilon (\overset{‾}{\nu })$$

where $\epsilon (\overset{‾}{\nu })$ is the molecular absorbance in $M^{-1}{\text{cm}}^{-1},\rho$ is the concentration, and b is the sample thickness. The absorbance is related to the imaginary component $\kappa (\overset{‾}{\nu })$ via $\epsilon (\overset{‾}{\nu })=\frac{4\pi \overset{‾}{\nu }\kappa (\overset{‾}{\nu })}{2.303\rho }$. An intensity measurement is given by the expression $I_{S}(\overset{‾}{\nu })=|P(\overset{‾}{\nu }){|}^{2}T_{S}(\overset{‾}{\nu })\text{exp}[-4\pi \overset{‾}{\nu }\kappa (\overset{‾}{\nu })b]$, where $P(\overset{‾}{\nu })$ is the incident amplitude and $T_{S}(\overset{‾}{\nu })$ is the transmission coefficient excluding absorption. To calculate absorption spectra, the background intensity is measured, $I_{0}(\overset{‾}{\nu })=|P(\overset{‾}{\nu }){|}^{2}T_{0}(\overset{‾}{\nu })$, where $T_{0}(\overset{‾}{\nu })$ is the net transmission coefficient for the instrumentation. The recorded absorbance $A(\overset{‾}{\nu })$ is obtained by taking the ratio:$A(\overset{‾}{\nu })={\text{log}}_{10}\left[\frac{I_{S}(\overset{‾}{\nu })}{I_{0}(\overset{‾}{\nu })}\right]=\frac{4\pi \overset{‾}{\nu }\kappa (\overset{‾}{\nu })b}{2.303}-{\text{log}}_{10}\left[\frac{T_{S}(\overset{‾}{\nu })}{T_{0}(\overset{‾}{\nu })}\right]$. Note that $T_{S}(\overset{‾}{\nu })=T_{0}(\overset{‾}{\nu })$ for homogeneous samples, but not for heterogeneous samples [20], [21].

## IV. Inverse Model

Two ambiguities arise when attempting to reconstruct a sample from absorbance images:

- The Beer-Lambert law (16) specifies two unknown components ρ and b that cannot be decoupled with a single measurement A.
- Variations in absorbance necessitate corresponding variations in n due to Kramers-Kronig (2) and (3), redistributing light in the final image and producing spectral aberrations.

Spectral aberration correction is an active area of research, particularly in biomedicine, where cellular features are near the wavelength of the incident light [19], [24], [30], [34], [35]. These methods rely on Mie scattering, which produces a similar distorted absorbance spectrum [36]. This includes iterative inversion for single spheres [30] and Mie-based corrections in tissue incorporating a phantom spectrum [19]. By far the most common method is piecewise linear baseline correction [18].

In this section, we demonstrate that chemical holography provides significantly better performance when using trained models for sample reconstruction.

### A. Layered Sample

**Fig. 3.**
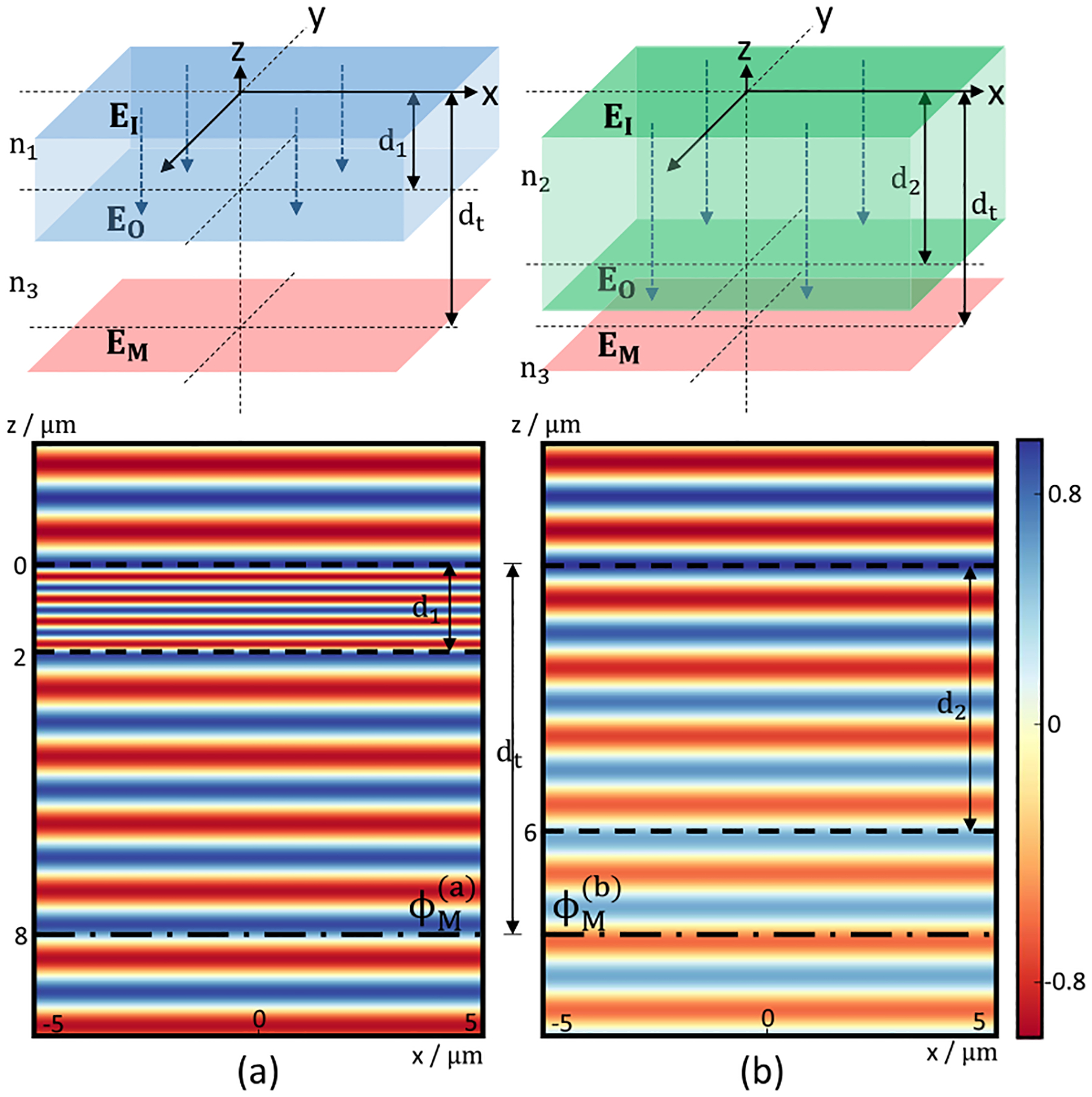
Different combinations of refractive index n and layer thickness d result in the same phase-shift for $E_{O}$. However, $E_{M}$ in (a) is different from $E_{M}$ in (b) due to light-path difference outside the upper layer. The wavelength λ=1.5μm, the complex refractive index for the middle layers are $n_{a}=3+0.05j$ and $n_{b}=1+0.05j$, and the thickness of the middle layers are $d_{1}=2\mu m$ and $d_{2}=6\mu m$, respectively.

The optical path length (OPL) of a non-absorbing layer ℓ can be defined as: $P_{\ell }=\text{nd}$, where $n(\overset{‾}{\nu })=m(\overset{‾}{\nu })+i\kappa (\overset{‾}{\nu })$ is the complex refractive index and d is its physical thickness. It is impossible to derive both n and d from a single intensity measurement. For example, an electric field $E_{I}$ propagating through two layered samples with the same effective OPL (Fig. 3), the field $E_{O}$ at the lower boundary would be identical. We demonstrate that it is possible to determine both n and d given $P_{\ell }$ with a phase-sensitive measurement. Assume the phase $E_{O}$ is $\phi_{O}$ in both cases (Fig. 3(a) and (b)). The phase shift from $E_{O}$ to field $E_{M}$ are given by: $\phi_{M}^{\left(a\right)}=\phi_{O}e^{in_{3}\left(d_{t}-d_{1}\right)},\phi_{M}^{\left(b\right)}=\phi_{O}e^{in_{3}\left(d_{t}-d_{2}\right)}$. While the phases are identical in the middle layer, the phase at z=8μm deviate due to the shift introduced by the geometric length. This provides another explicit constraint for calculating n and d.

We construct an optimization algorithm to fit m,d, and κ by minimizing a cost function. Consider a middle layer with unknown material properties embedded in air corresponding to the following parameters: (a) effective OPL, (b) distance between the upper boundary of the sample and detector $d_{t}$, (c) incident plane wave $E_{I}$, and (d) measured field $E_{M}$. A finite number of m and d pairs exist in the range $m\in \left[m_{\text{min}},m_{\text{max}}\right]$ with precision ϵ given by: $N=\frac{m_{\text{max}}-m_{\text{min}}}{\epsilon }$. The resulting cost function is: $\left.\text{min}(\Theta )=\text{min}\left(|\phi_{f}\left(m_{i},d_{i}\right)\right)-\phi_{0}|\right)$, where $m_{i}d_{i}=P$ for i∈(0,N]. Since the impact of the extinction coefficient on phase is negligible, the same optimization is repeated for κ after m and d are determined.

### B. Mie Scattering

From (13), the inverse Mie problem [37] is the solution to a linear system:

(17) $$\left[\begin{array}{cccc} H_{0}\left(r_{0}\right) & H_{1}\left(r_{0}\right) & \cdots & H_{N}\left(r_{0}\right)\\ H_{0}\left(r_{1}\right) & H_{1}\left(r_{1}\right) & \cdots & H_{N}\left(r_{1}\right)\\ \vdots & \vdots & \ddots & \vdots \\ H_{0}\left(r_{N}\right) & H_{1}\left(r_{N}\right) & \cdots & H_{N}\left(r_{N}\right) \end{array}\right]\left[\begin{array}{c} B_{0}\\ B_{1}\\ \vdots \\ B_{N} \end{array}\right]=\left[\begin{array}{c} E_{s}\left(r_{0}\right)\\ E_{s}\left(r_{1}\right)\\ \vdots \\ E_{s}\left(r_{N}\right) \end{array}\right]$$

where

(18) $$H_{t}(r)=\sum_{t=0}^{\infty }(2t+1)i^{t}h_{t}^{\left(1\right)}(\text{kr})P_{t}(\text{cos}\theta )$$

and $E_{s}(r)$ is the scalar value for the scattered field at spatial location r. This matrix H has a condition number $c\gg 10^{20}$, so analytical solutions are limited to low-contrast (n≈1) and non-absorbing (κ=0) materials. [38], [39] Artificial neural networks (ANNs) have been proposed as an effective form of regularization [40], [41] for more general materials. We design a multi-layer ANN to solve the inverse Mie problem and compare the results between absorbance and holographic infrared imaging.

The Mie scattering model is radially symmetric [42], so there is no angular dependence in the far field (15):

(19) $$E_{F}(r)=\sum_{t=0}^{\infty }B_{t}(\lambda ,n,a)\frac{e^{\left[i(\text{kr}-t\pi /2)\right]}}{\text{ikr}}P_{t}\left(\text{cos}\theta_{\|}\right)$$

The near field is calculated using the Hankel transform as a radial analog of the FFT [43], [44]: $E_{F}(r)\overset{{ℋ}^{-1}\;}{\to }E_{0}(r)$, where ${ℋ}^{-1}$ represents the order-0 inverse discrete Hankel transform.

**Fig. 4.**
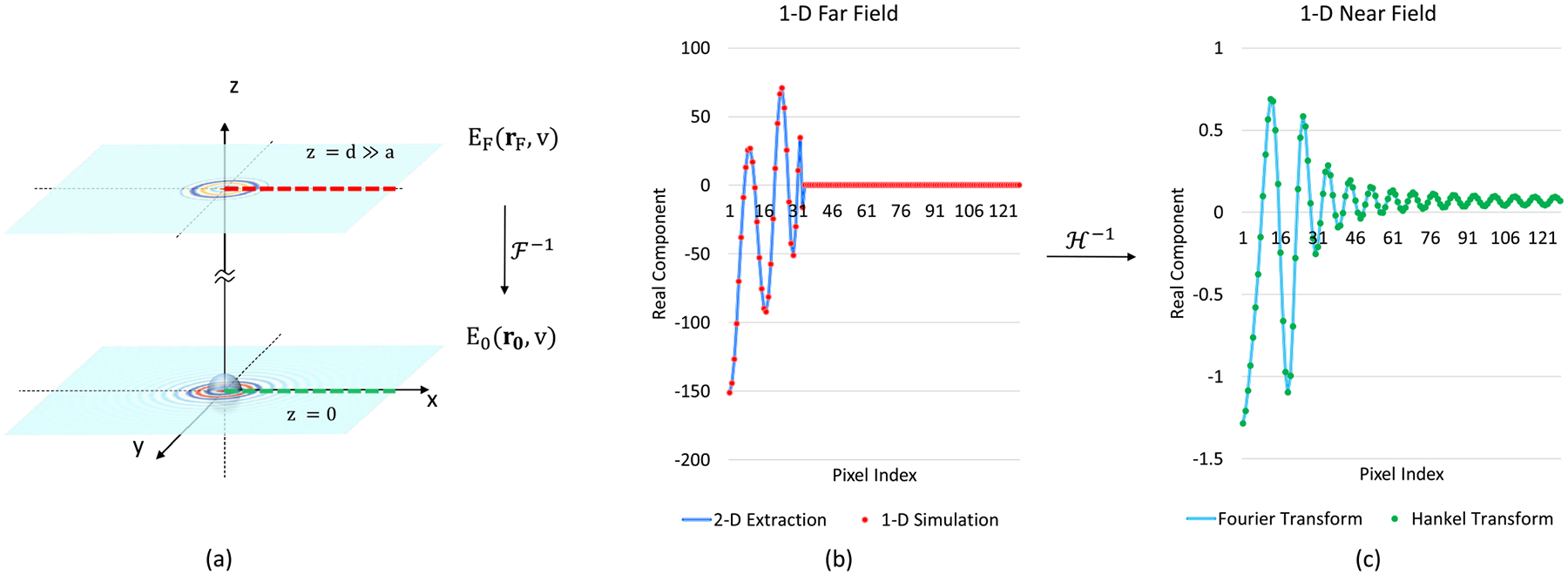
Using discrete hankel transform to get the 1D representation of the near-field simulation. (a) The 2-D far-field simulation. The corresponding 1D representation is marked as thick dashed lines (red dashed line for the far field in the reciprocal domain and green dashed line for the near field in the spatial domain). (b) The 1D representation of the far field in the Fourier domain, the blue line is the 2D extraction, and the red dots are 1D simulation results. (c) The 1D representation of the near field in the spatial domain, the blue line is extracted from the 2D Fourier transform of a 2-D simulation, and the green dots are acquired from discrete hankel transform of a 1D far-field simulation. The material properties of the simulated sphere are: a=2μm,m=1.4,κ=0.02. The resolution and the FOV of the images are 256 × 256 and 32μm×32μm, respectively.

Fig. 4 shows the simulated 1D representation of Mie scattering for a sphere converted from a 1D far field simulation using the discrete inverse Hankel transform, the results of which are identical to that of 2-D models. Similar to the Fourier transform, an order-v Hankel-transform converges with increasing FOV in the spatial domain or a higher resolution in the reciprocal domain.

**Fig. 5.**
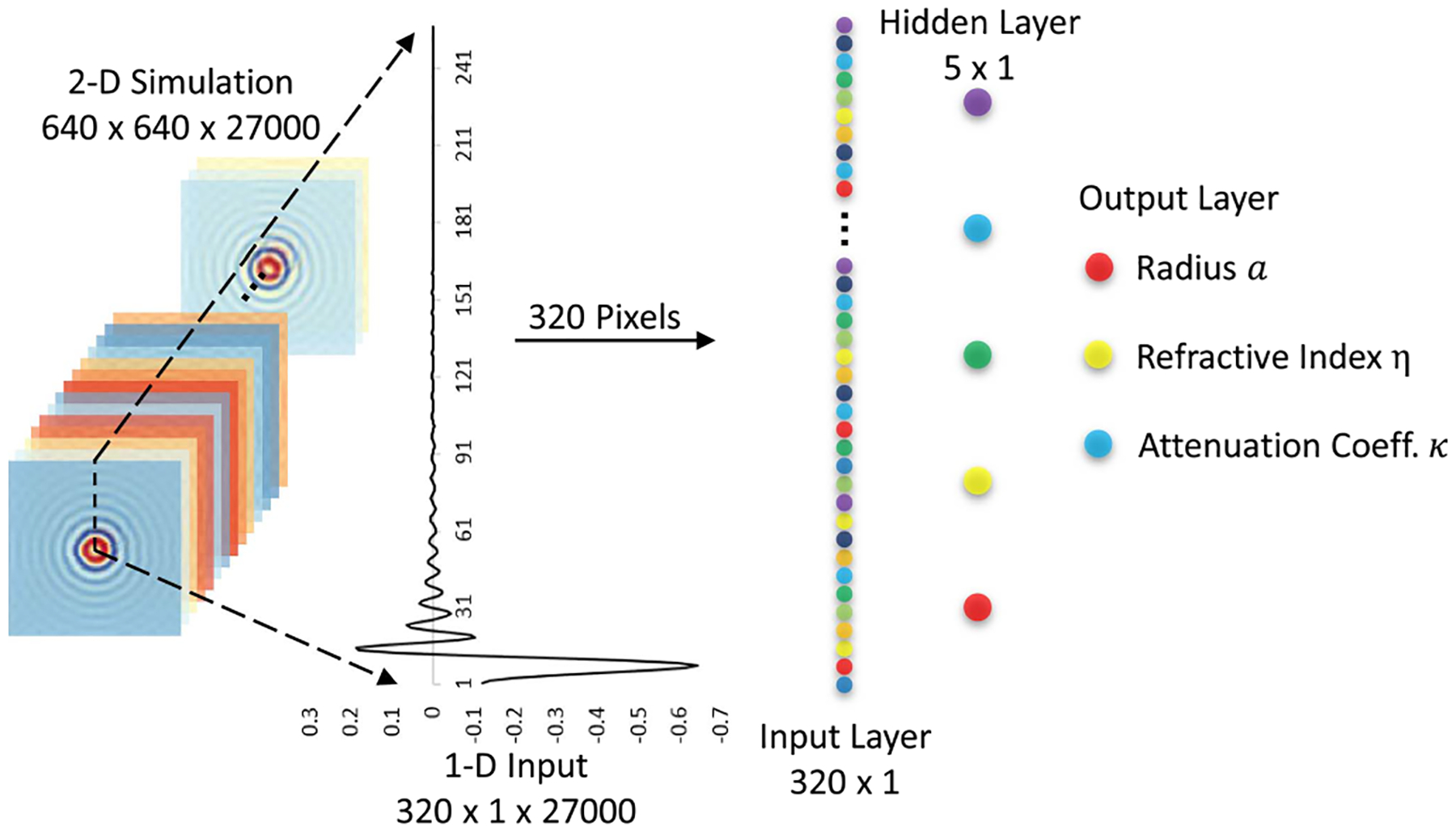
One-layer artificial neural network used for solving the inverse Mie problem.

We use a single-layer ANN with 5 hidden nodes (Fig. 5) to solve the ill-conditioned inverse Mie problem in 1D to demonstrate the merits of phase-sensitive measurements. Random sampling a Glorot Normal Distribution [45] initializes the weights for the hidden layer with zero mean and standard deviation: $\sigma =\sqrt{\frac{2}{N_{i}+N_{o}}}$, where $N_{i}=320$ is the number of input units in the weight tensor and $N_{o}=5$ is the number of output units. The activation functions for the hidden and output layers are hyperbolic tangent and linear functions, respectively. Given a 1D vector representing the scattered field as the input to the trained ANN, the output is denoted by three units corresponding to a radius a sphere with refractive index m and extinction coefficient κ.

## V. Chemical Holographic Imaging Model

**Fig. 6.**
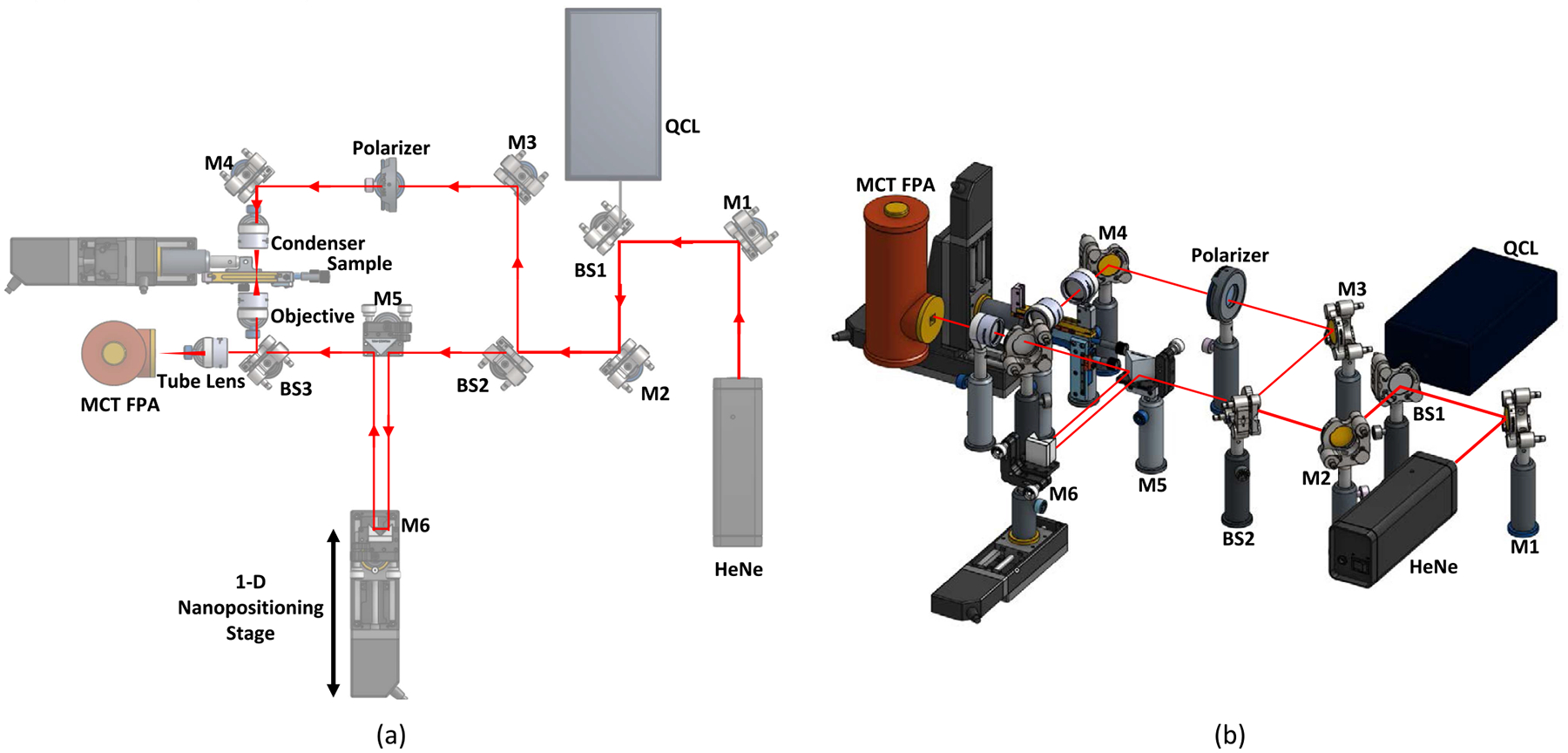
Chemical holographic imaging system design showing the infrared optical path (a) and 3D rendering (b).

The proposed instrumentation combines an interferometer with a high-resolution microscope and quantum cascade laser source (Fig. 6). A HeNe laser is used for alignment and visible feedback. The QCL emission is divided into sample and reference beams using a (50/50) beamsplitter (BS2). A polarizer is placed between guide mirrors M3 and M4 to compensate for intensity differences between both beams. The sample beam is focused through a low-NA condenser to fill the field of view (FOV). The transmitted field is collected by an objective and projected onto another beamsplitter (BS3 in Fig. 6(a)). The reference beam is reflected twice between a triangular (M5) and prism mirror (M6) before reaching BS3, where the two beams are recombined. A tube lens converts the intermediate image to the spatial domain and focuses onto an MCT detector.

A nanopositioning stage controls the optical path difference (OPD) between the sample and reference arms to collect an interferogram image. A second 2D nanopositioning stage moves the sample to collect a mosaic for images. The raw interferogram is processed in real-time to reconstruct a complex image of the electromagnetic field.

We define the incident field polarization using the unit vector $\overset{ˆ}{e}=\frac{E}{\sqrt{I}}$ such that ${\overset{ˆ}{e}}^{H}\overset{ˆ}{e}=1$. Light transmitted through the sample is characterized by a scalar electric field $E_{R}^{m}(r,\overset{‾}{\nu })\in C$ (Fig. 6), where $r=[x,y{]}^{T}$ is a point in the detector image. The reference beam is denoted by $E_{R}(r,\overset{‾}{\nu })$. Light from the objective and reference arms interfere and the intensity $I(r,\overset{‾}{\nu })$ is recorded.

An interferogram is collected by adjusting the reference arm OPL by $\Delta =\left[\Delta_{1},\Delta_{2},\ldots ,\Delta_{M}\right]$, where the objective and reference arms are equal at Δ=0. A collimated reference beam applies a uniform phase-shift $E_{R}^{m}(r,\overset{‾}{\nu })=E_{R}(r,\overset{‾}{\nu })e^{i\left(\frac{2\pi }{\lambda }\right)\Delta_{m}}$, where m∈[1,M]. The instrument output is divided into (a) the objective field $E_{\text{obj}}$ and (b) reference field $E_{\text{ref}}$. In our simulation, $E_{\text{obj}}$ is the output of the far-field Mie scattering model, and $E_{\text{ref}}$ is a series of plane waves with constant phase-shift. An M-images interferogram is generated by: $I^{\left(m\right)}=\left(E_{\text{obj}}+\right.{\left.E_{\text{ref}}^{\left(m\right)}\right)}^{H}\left(E_{\text{obj}}+E_{\text{ref}}^{\left(m\right)}\right),m\in [1,M]$, where $I^{\left(m\right)}$ denotes the $m_{\text{th}}$ intensity image.

The intensity measured at the detector for each discrete path length difference $\Delta_{m}$ is:

(20) $$I^{m}\left(r,\bar{\nu }\right)={\left|E_{S}\left(r,\bar{\nu }\right)\right|}^{2}+{\left|E_{R}\left(r,\bar{\nu }\right)\right|}^{2}\;+E_{S}\left(r,\bar{\nu }\right)E_{R}^{*}\left(r,\bar{\nu }\right)e^{-\text{ik}\Delta_{m}}\;+E_{S}^{*}{\left(r,\bar{\nu }\right)}_{R}\left(r,\bar{\nu }\right)e^{\text{ik}\Delta_{m}}$$

where $k=2\pi \overset{‾}{\nu }$, and * denotes the complex conjugate. Assuming that measurements are performed at uniform intervals $\left(\left(\Delta_{m+1}-\Delta_{m}\right)=\delta \right)$, intensity differences form a system of linear equations:

(21) $$\begin{array}{l} \left[\begin{array}{c} I^{2}\left(r,\bar{\nu }\right)-I^{1}\left(r,\bar{\nu }\right)\\ \vdots \\ I^{M}\left(r,\bar{\nu }\right)-I^{M-1}\left(r,\bar{\nu }\right) \end{array}\right]=\left[\begin{array}{cc} e^{-\text{ik}\Delta_{1}} & e^{\text{ik}\Delta_{1}}\\ \vdots & \vdots \\ e^{-\text{ik}\Delta_{M-1}} & e^{\text{ik}\Delta_{M-1}} \end{array}\right]\;\times \left[\begin{array}{c} \left(e^{-\text{ik}\delta }-1\right)E_{S}\left(r,\bar{\nu }\right)E_{R}^{*}\left(r,\bar{\nu }\right)\\ \left(e^{+\text{ik}\delta }-1\right)E_{S}^{*}\left(r,\bar{\nu }\right)E_{R}\left(r,\bar{\nu }\right) \end{array}\right] \end{array}$$

or simply as $D(r,\overset{‾}{\nu })=Pu(r,\overset{‾}{\nu })$, where P is known. Experimental data is inverted to calculate the interferometric cross-term (two per pixel position r) using the Moore-Penrose inverse:

(22) $$\left[\begin{array}{c} u(r,\overset{‾}{\nu })\\ u^{*}(r,\overset{‾}{\nu }) \end{array}\right]=u(r,\overset{‾}{\nu })={\left(P^{†}P\right)}^{-1}P^{†}D(r,\overset{‾}{\nu }),$$

where † denotes the Hermitian conjugate. Because of the collimated reference beam, the phase is constant across the image, and $\left|E_{R}(r,\overset{‾}{\nu })\right|$ can be measured by blocking light from the sample arm. Then (22) to find $E_{S}$ by:

(23) $$E_{S}(r,\overset{‾}{\nu })=\frac{u(r,\overset{‾}{\nu })}{\left|E_{R}(r,\overset{‾}{\nu })\right|\left(e^{-\text{ik}\delta }-1\right)}$$

## VI. Results

We examine the performance of our inversion approaches for layered and Mie models to demonstrate the merits of holographic measurements. This includes the performance of the machine learning models trained with complex data tested against those trained solely with traditional intensity.

### A. Phase Reconstruction

**Fig. 7.**
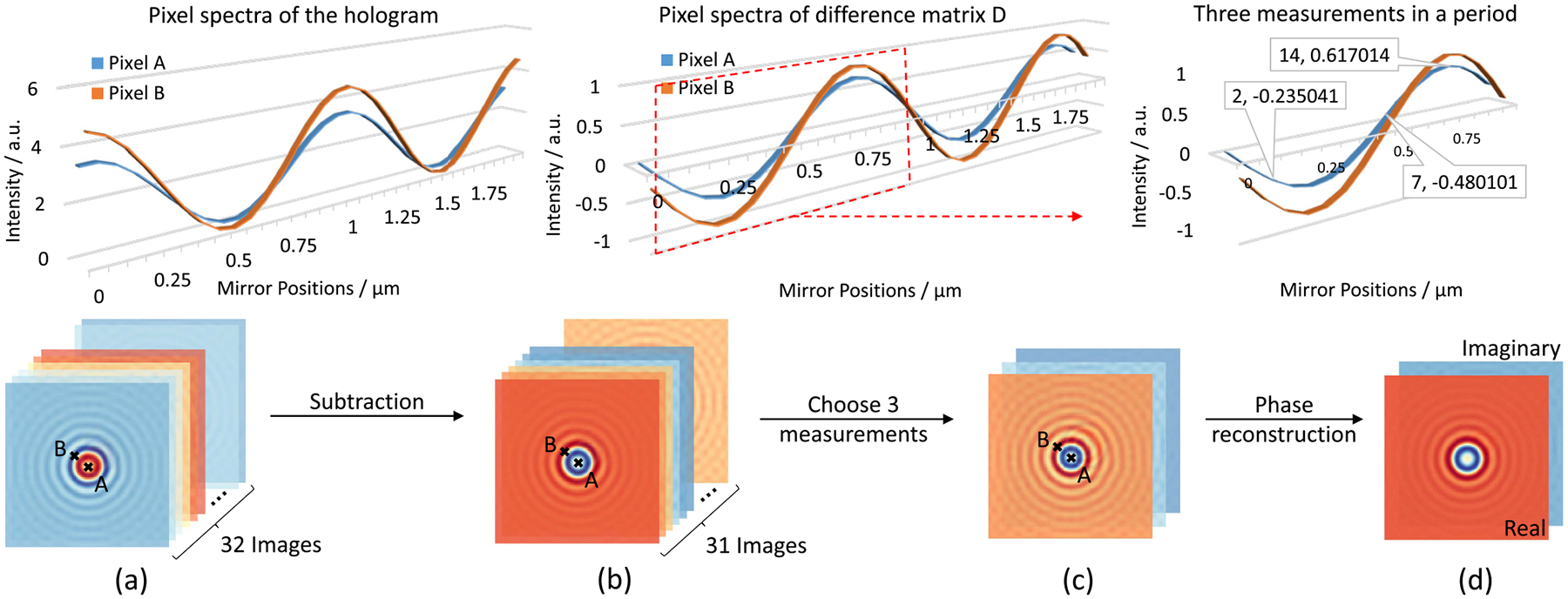
The workflow of the complex-field reconstruction for chemical holography. 3D-line plots on the top row are the pixel intensity spectra corresponding to pixel A and pixel B marked in (a), (b) and (c), respectively. (a) 32 intensity images in the simulated hologram with total light path difference ${(\Delta }_{\text{total}}=2\mu m)$ equal to twice the wavelength (λ=1μm). (b) 31 difference images acquired by subtracting the adjacent images in (a). (c) Three measurements (2nd, 7th, and 14th measurement) down-sampled from (b). (d) Reconstructed field with real and imaginary components.

A hologram of a sphere with radius a=1μm and complex refractive index n=1.5+i0.03 is simulated using a Mie scattering model (Fig. 7(a)). The incident wavelength is 1μm, and the total light path difference is 2μm. The interferogram contains 32 intensity images (128 × 128) to measure the sinusoidal waveform (Fig. 7 top row). The field of view (FOV) is 16μm×16μm. Pixel A is located at the center $\left(\left(x_{A},y_{A}\right)=(64,64)\right)$ and pixel B is 0.25μm from the sphere surface $\left(\left(x_{B},y_{B}\right)=(54,54)\right)$. Note that the amplitude at B is higher A, which is expected due to stronger scattering near the sphere surface [46].

**Fig. 8.**
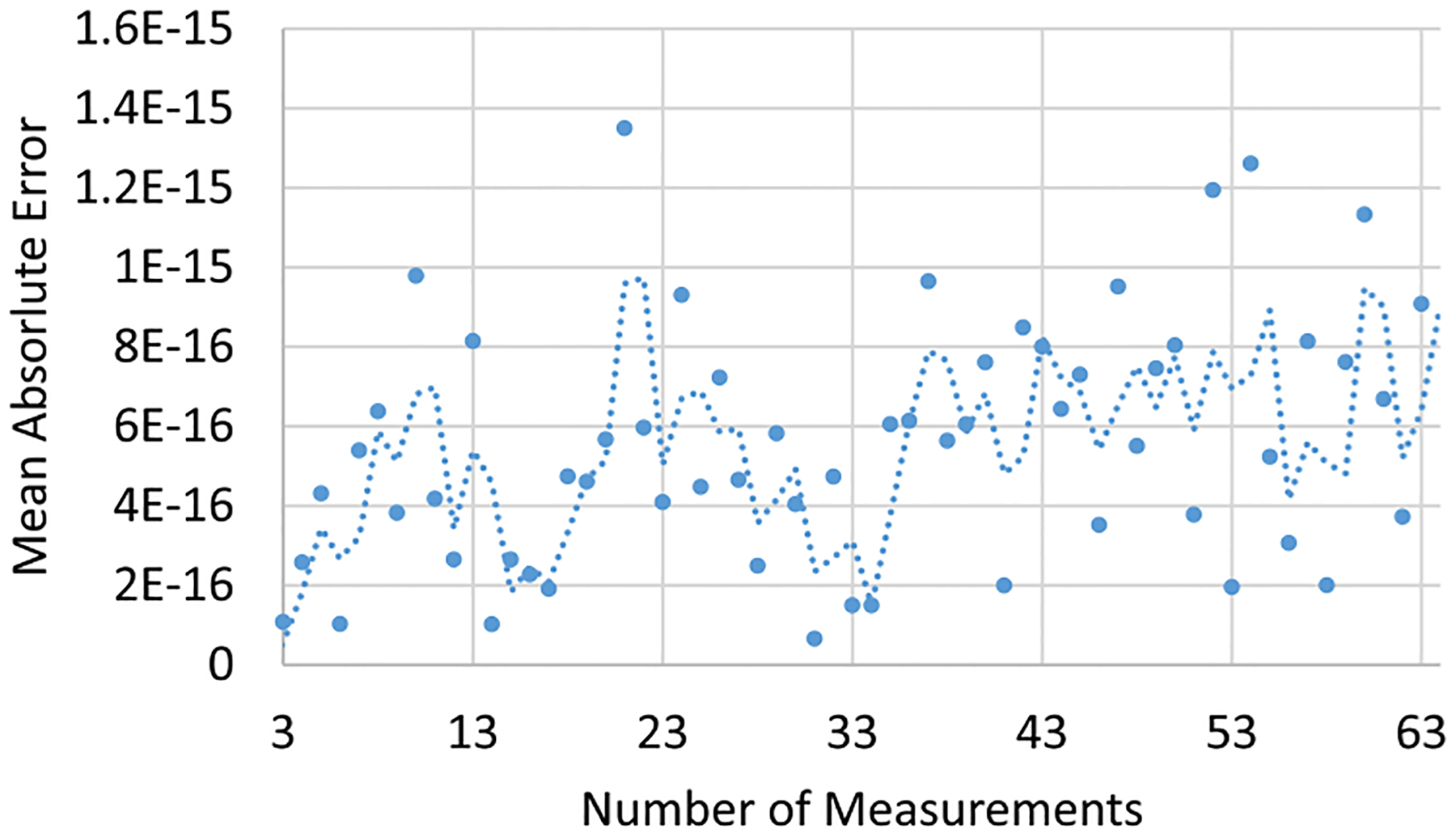
Scatter plot of the mean absolute error along the number of measurements used for the phase reconstruction. In the simulation, the error is low for all number of measurements since no external noise is induced. The dashed line denotes a size-2 window average.

Fig. 7(b) shows the intensity images forming D acquired by subtracting consecutive images. The interferogram starts at zero path difference $\left(\Delta_{1}=0\right)$ producing two sinusoidal waves. The resulting hologram is calculated by fitting these waveforms to sinusoidal functions at the incident wavelength to calculate the amplitude and phase. We present 3 representative images from the first half of the interferogram (Fig. 7(c)), resulting in a reconstruction error on the order of $10^{-16}$ (Fig. 8).

We first look at numerical error resulting from simulation and reconstruction in the absence of noise using our approach (Fig. 8). In this case the error is relatively low $\approx 10^{-15}$ since the dominant error is from floating point calculations during the matrix inversion. Practical measurements include ambient, thermal, and phase noise induced by mechanical vibrations, which are discussed next.

**Fig. 9.**
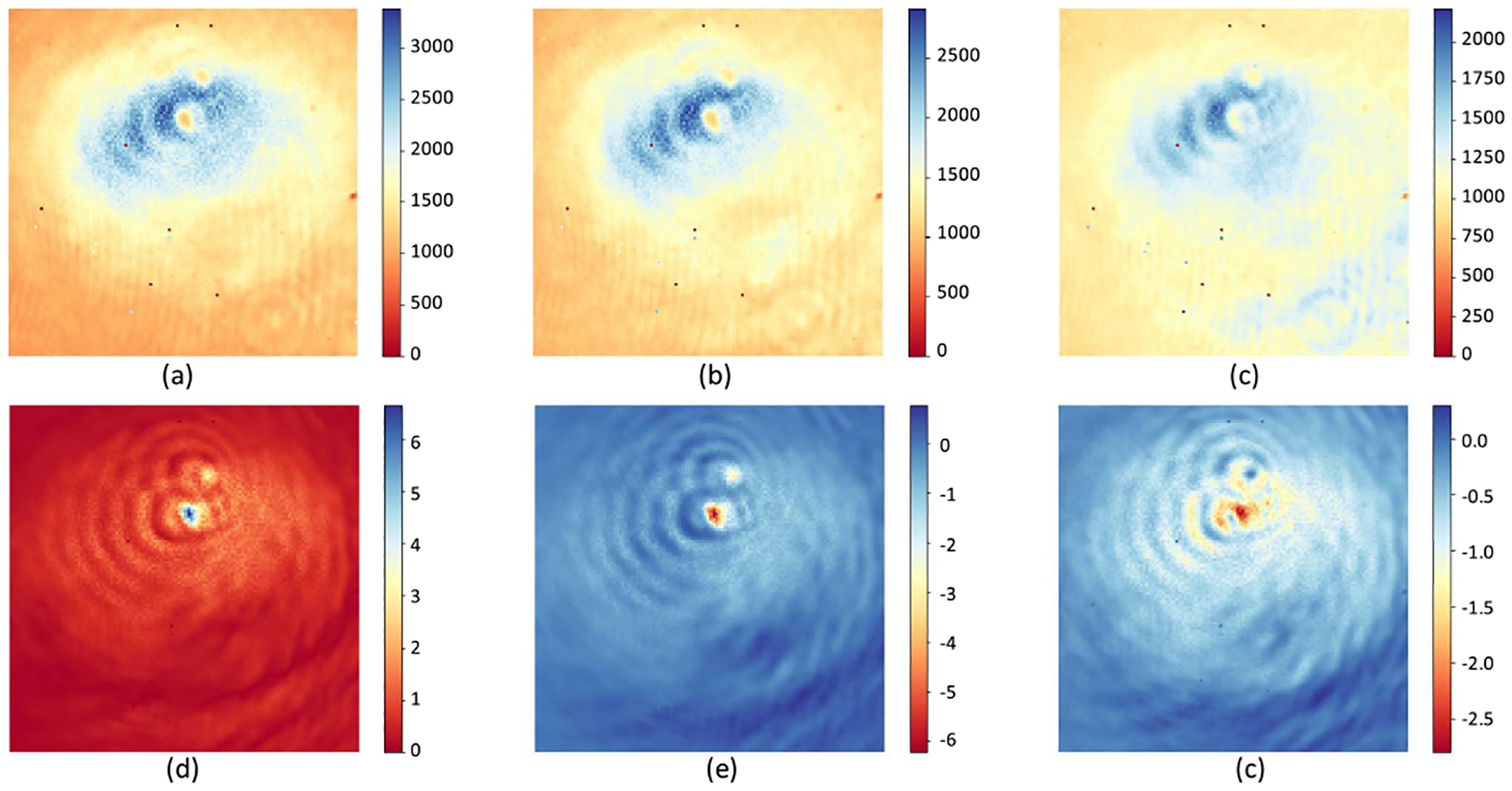
Reconstructed phase images using CHIS. (a)–(c) Are the first three images in the hologram that used for reconstruct the phase. (d) The absolute image of the reconstructed field, (e) the real component of the filed, (f) the imaginary component of the filed.

Fig. 9 presents experiments using two PMMA (polymethyl methacrylate) spheres (20μm and 10μm diameters) imaged by the system with $1250{\text{cm}}^{-1}(8\;\mu m)$ emission from the QCL. The hologram contains 200 images with a 40 nm step-size to cover an optical path difference of 8μm. Only the first 3 images (Fig. 9(a)–(c)) are required for reconstruction. It is clear that the reconstructed images have more visible features compare to the original hologram, such as the shape of the spheres and the fringing artifacts caused by scattering.

### B. Layered Model Inversion

**Fig. 10.**
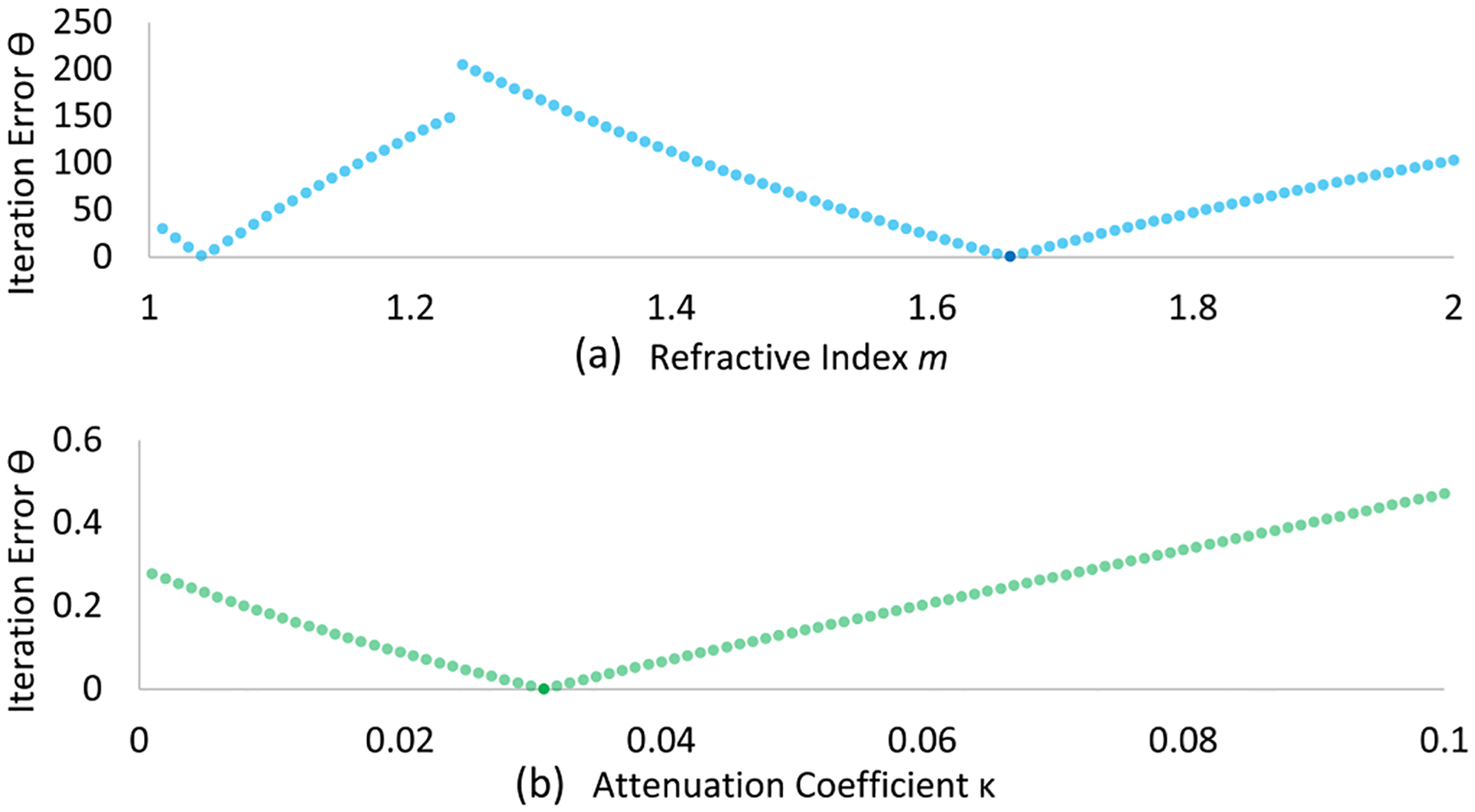
The iteration error along (a) refractive index m and (b) extinction coefficient κ.

A 3-layer model is simulated, where the middle layer (target) is embedded in air with $m_{\text{gt}}=1.65,d_{\text{gt}}=1.67$, and $\kappa_{\text{gt}}=0.03$. The measured field $E_{M}$ is simulated using the described coupled-wave calculation. Fig. 10 shows the reconstruction error along m and κ, respectively.

We benchmark reconstruction in the presence of noise by assuming Gaussian distributed noise with zero mean at the detector. If the layered sample is uniform in shape, (i.e., the interfaces are flat, and the incident plane wave is impinging the sample perpendicularly) then the field measured at the detector can be averaged across all the pixels.

**Fig. 11.**
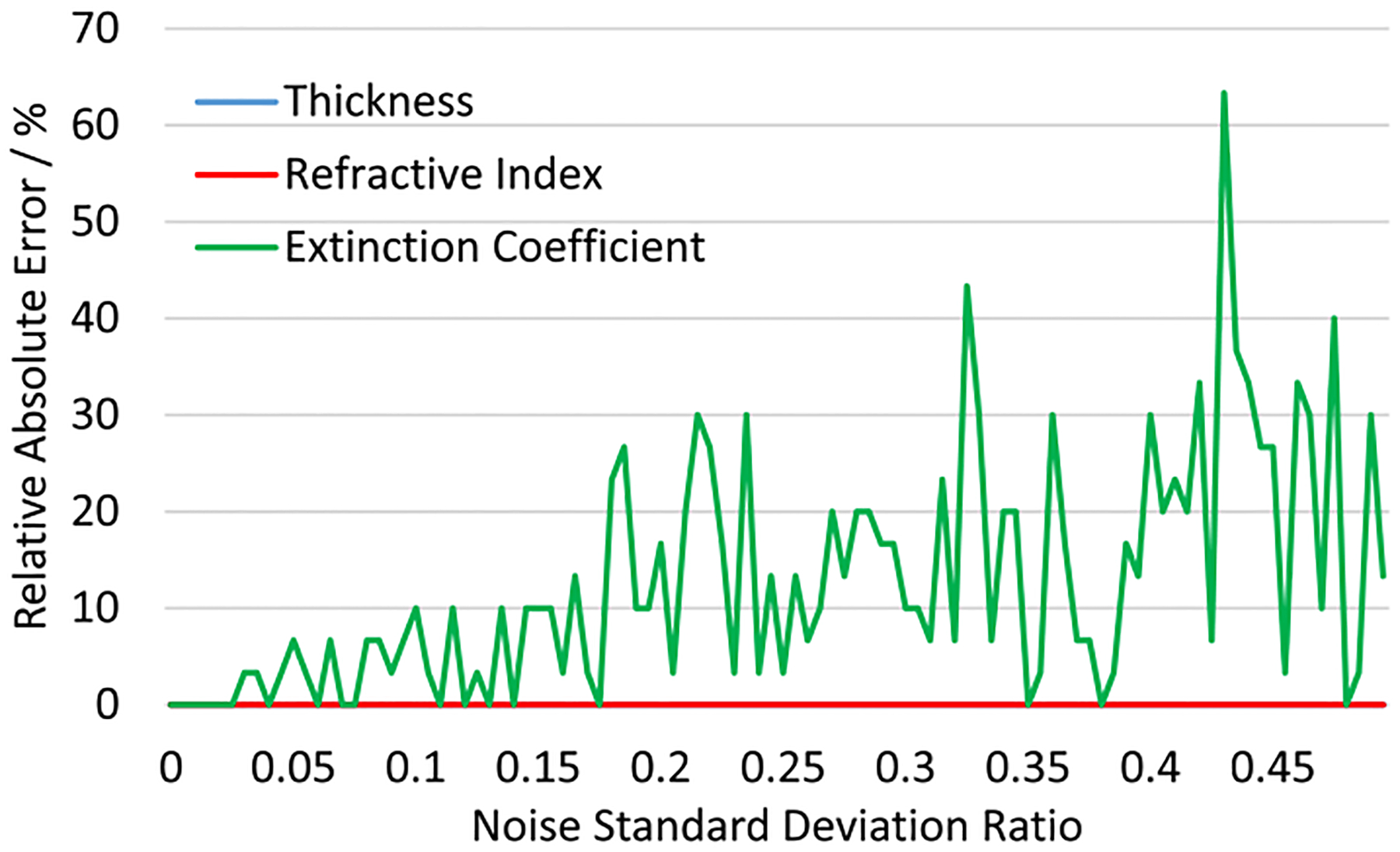
Relative absolute error with increasing standard deviation the Gaussian distributed noise when solving the inverse model.

Experiments are done by repeating the inversion with increased noise standard deviation. As shown in Fig. 11, the optimization retrieves the layer thickness d and refractive index n consistently. However, the error in the extinction coefficient κ increases, since its magnitude is much smaller.

### C. Mie Scattering Inversion

As described in previous sections, the inversion of the Mie Scattering problem is ill-conditioned and highly noise sensitive. We instead approximate the inversion relationship of the system using a neural network.

#### 1) Artificial Neural Network:

To ensure the ANN is trained with low bias and variance, 27000 complex 1D representations (320 × 2) of the scattered field are generated by the 1D far-field Mie scattering model (Fig. 4) with different material properties for the simulated spheres. Since the reconstructed field contains complex values, the real and imaginary components of the complex values are separated as two channels for the input (hence 320 × 2). For each sphere, the feature vectors has 30 entries uniformly distributed within their predefined range. Specifically, the radius $a\in \left[1,2\right]\mu m$, the refractive index m∈[1.1,2.0], and the extinction coefficient κ∈[0.01,0.05].

Before the training, the entire data set is shuffled and split into training and testing sets with a ratio of 9 : 1. The training set is further divided into training and validation sets. The validation set contains 20% of the randomly chosen training data for each epoch, results with 19440 samples for training and 4860 samples for validation. The training process contains 400 epochs (i.e., the training-cross-validation process is repeated for 400 times) and the batch size is set to 200. Meanwhile, feature scaling is applied to the extinction coefficient set due to feature scale difference.

A second ANN model is trained on a traditional intensity measurement of the same simulations. The intensity ANN is trained with an identical network architecture (Fig. 5), and with the same number of training and testing samples. The intensity data is acquired by applying (20) to the complex 1D vectors. The square root of the intensity is used to regularize the range of these two data sets and improve performance.

Table I shows the performance of the complex and intensity ANNs summarized from 30 repetitive trails. The complex ANNs perform better than intensity ANNs, which can be partially explained by the fact that the additional phase information existing in the complex training set is no longer available for the intensity training set (during the transaction from complex to intensity). Moreover, the performance of the ANN is further increased by adding additional layers and hidden units. Table II shows the performances of an ANN trained with the same data sets.

The relative root-mean-square error (RMSE) $\epsilon_{R}$ is calculated by:

(24) $$\epsilon_{R}=\frac{\sum_{m=1}^{M}\frac{\sqrt{{\left(y_{\text{pred}}^{\left(m\right)}-y_{\text{test}}^{\left(m\right)}\right)}^{2}}}{y_{\text{test}}^{\left(m\right)}}}{M}$$

where $y_{\text{pred}}$ is the prediction given by the ANN model and $y_{\text{test}}$ is the ground truth (label) of the corresponding sample. The total number of test samples is denoted by M=4860.

#### 2) Synthetic Noise Test:

To better examine the impact of the phase information recovered by chemical holography, the performance of these two types of ANNs (with 5 hidden units) are tested on various testing sets, including synthetic data sets with unstructured and structured noise, specifically, we add Gaussian noise as unstructured noise and two other sources of structured noise (bandpass filtering effect and multi-sphere scattering) into the data set.

**Fig. 12.**
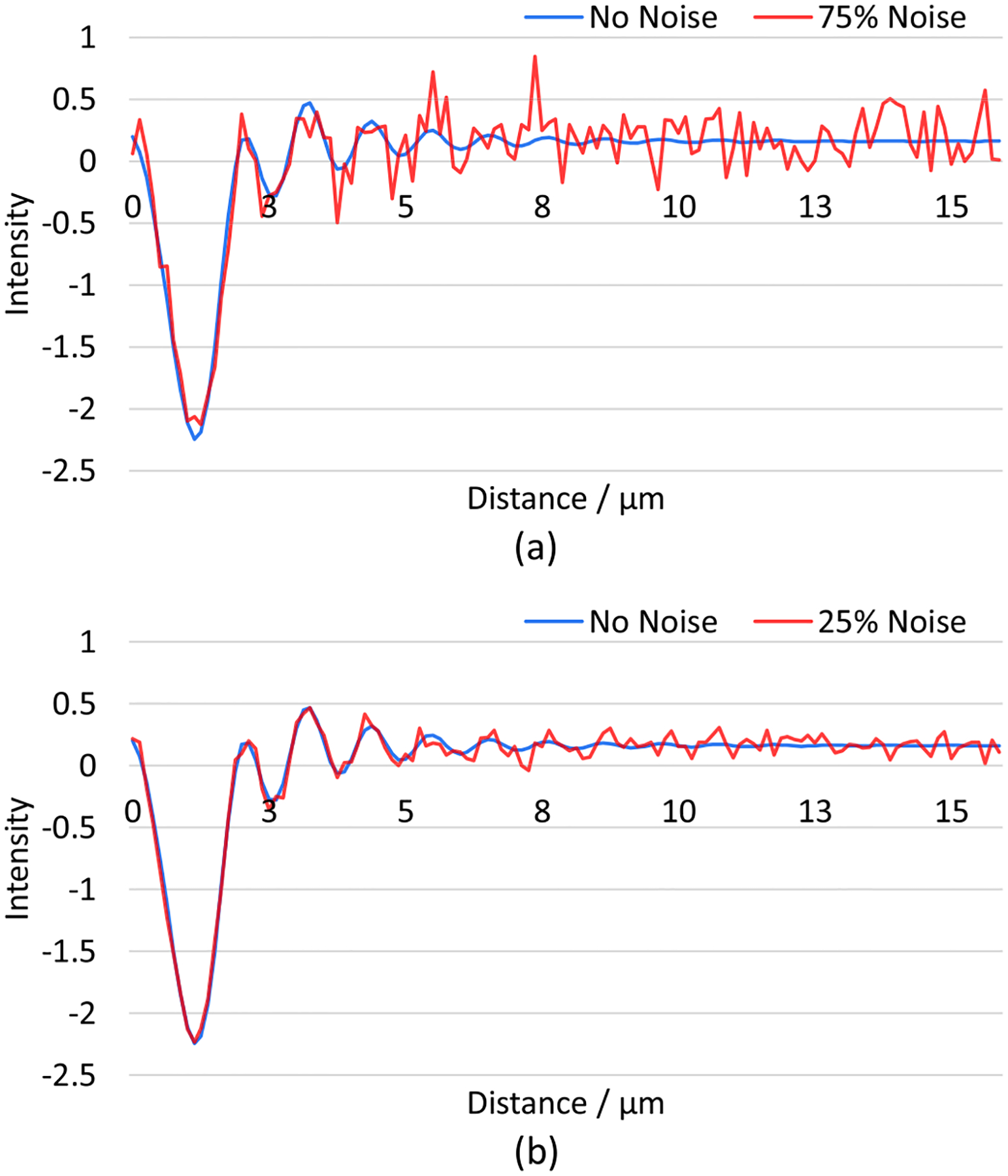
Two samples from the synthetic noisy data set. The material properties for the simulated sphere are a=2,m=1.5,κ=0.01. The resolution and FOV are 128 × 1 and 16μm×16μm, respectively. (a) 1D far-field simulation with 25% of Gaussian noise with a standard deviation of 0.25 and 0 mean. (b) 1D far-field simulation with 75% of total noise. Only real components are shown.

We assume that the combined effect of all noise in the system is Gaussian distributed with a fixed standard deviation and zero mean. Besides the three original features of the sphere material properties, the training and test data is enlarged by adding the noise level as another feature. To better examine the sensitivity of two ANN models with respect to synthetic noise, we decrease the number of features for the sphere properties down to 10 for each feature, and increase the number of noise levels up to 100, ranging in [1, 100]%, which results in 100,000 total number of samples for training and test. Specifically, the noise for real and imaginary components of the field are drawn independently within the same distribution. Also, the intensity training and test set are calculated from the complex set. Fig. 12 shows two samples (real part) from the synthetic noisy data with noise levels at 75% and 25%, respectively.

**Fig. 13.**
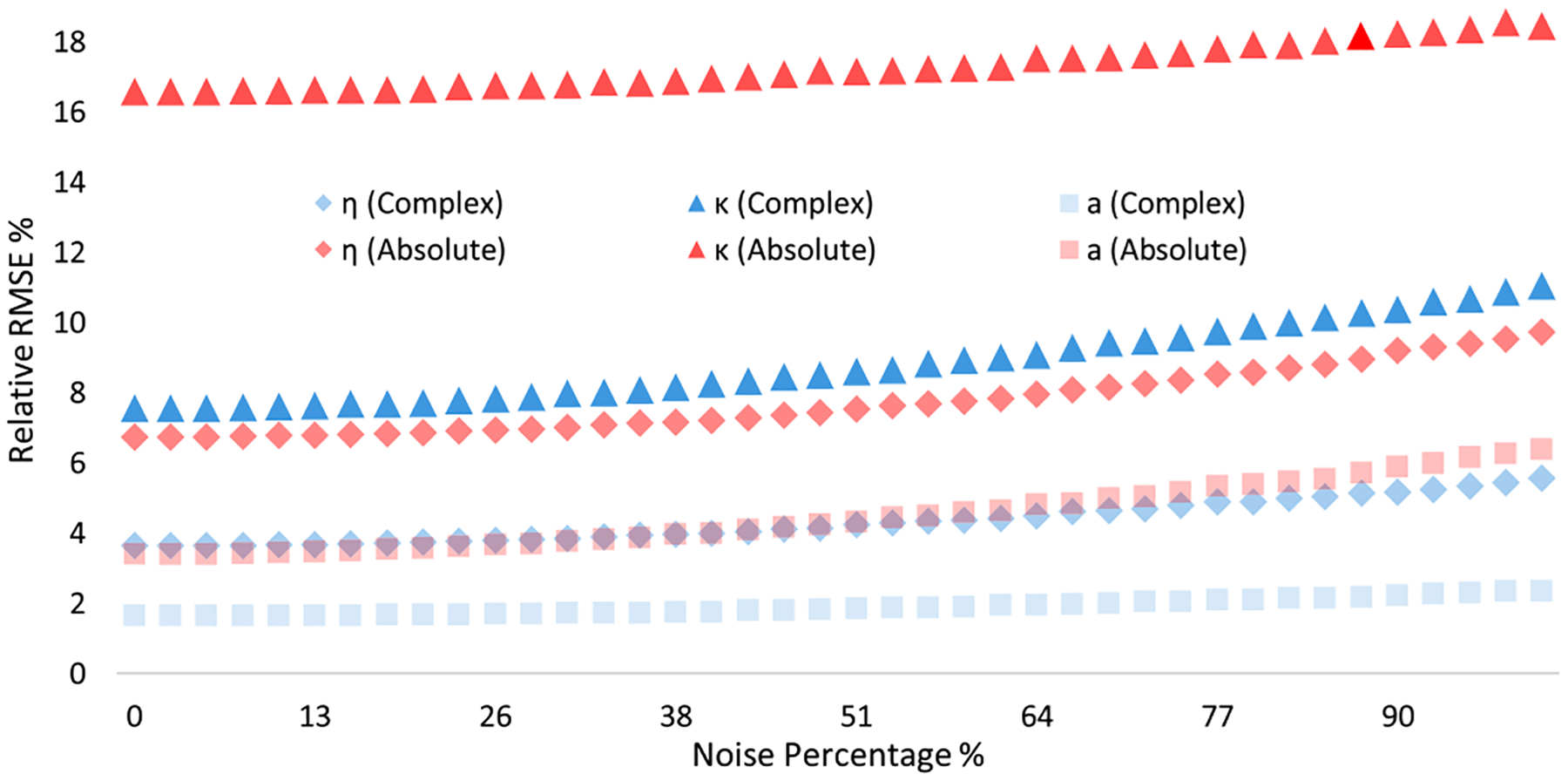
Relative RMSE plots for refractive index m, extinction coefficient κ, and radius a tested on 100 synthetic noisy data sets.

The training and test process for synthetic noisy data follows the same pipeline described above. With another implicit feature: noise level added into the training set, the performances of the ANNs are expected to decrease slightly. The point here, however, is to inspect the overall performances of those two types of ANNs with respect to noise levels. Fig. 13 shows the prediction accuracy of complex ANN and intensity ANN tested on 40 different test sets with different noise levels. It is obvious that complex ANN outperforms intensity ANN under all noisy conditions.

#### 3) Bandpass Filtering Noise:

In the 2-D case, the process of bandpass filtering can be described by the principle of Fourier optics. Specifically, the high-frequency and low-frequency components of the field going into the objective is blocked by the upper and lower cutoff frequencies given by: $f_{u}=\frac{NA_{\text{out}}}{\lambda }$ and $f_{l}=\frac{NA_{\text{in}}}{\lambda }$.

**Fig. 14.**
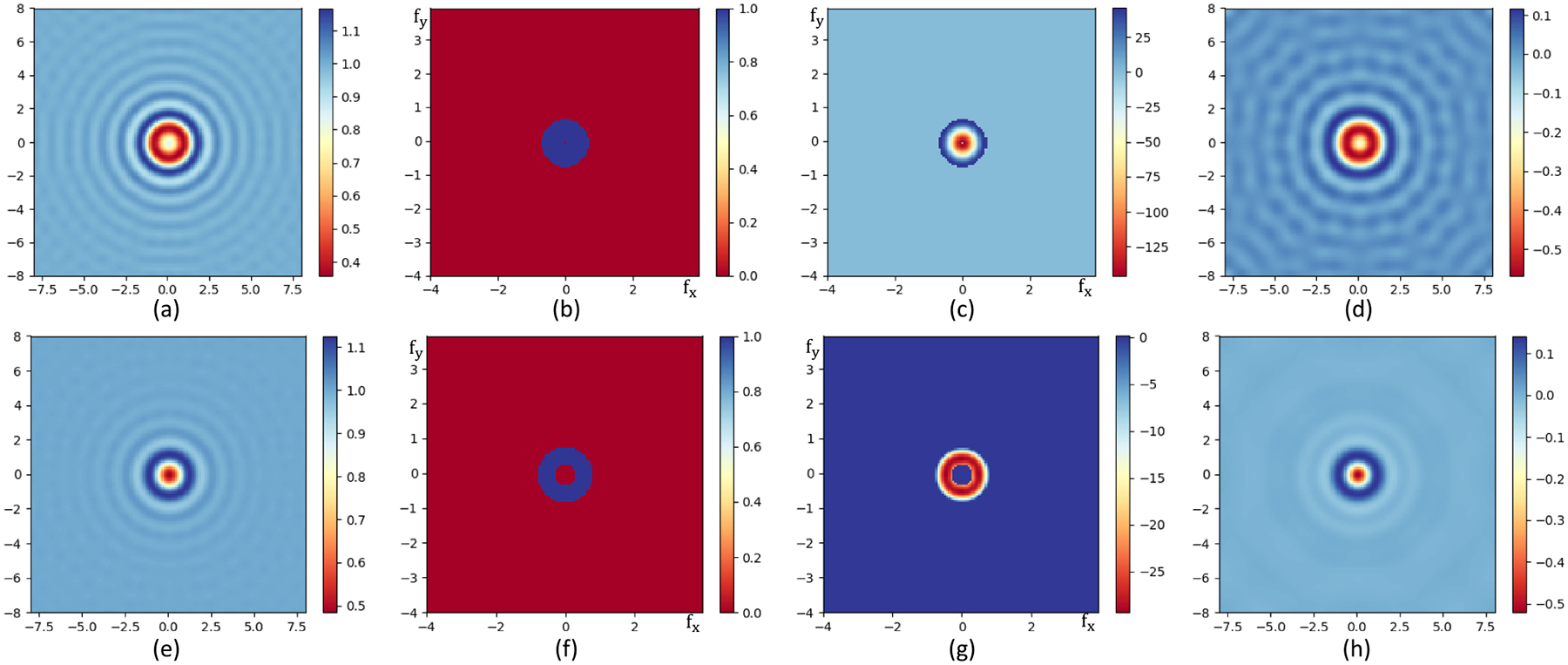
Bandpass filtering for two difference spheres. The field of view of the images is 16μm×16μm, the image resolution is 128 × 128. The radius of the spheres is a=1μm and the incident wavelength is λ=1μm. The material properties of these two spheres are $m_{1}=1.5,\kappa_{1}=0.05$ for (a)–(d), and $m_{2}=1.25,\kappa_{2}=0.01$ for (e)–(g), respectively. (a) Total field for the first sphere before bandpass filtering. (b) Bandpass filter simulating a Cassegrain objective with center obscuration $\left(NA_{\text{in}}=0.0\right)$ and back aperture $NA_{\text{out}}=0.7$, plotted in the Fourier domain. (c) The filtered field in the Forier domain. (d) Filtered field transformed back into spatial domain. (e) Total field for the second sphere before bandpass filtering. (f) Bandpass filter simulating a refractive objective without center obscuration $\left(NA_{\text{in}}=0.3\right)$ and back aperture $NA_{\text{out}}=0.8$, plotted in the Fourier domain. (g) The filtered field in the Forier domain. (h) Filtered field transformed back into spatial domain.

As shown in Fig. 14, the complex field is transformed into the frequency domain by performing a forward Fourier transform F(u,v)=ℱ[f(x,y)]. For an image with a FOV denoted by S and a resolution of R, The frequency-domain representation of the objective is a concentric circle with inner and outer radius specified by the center obscuration $NA_{\text{in}}$ and the back numerical aperture $NA_{\text{out}}$ of the objective:

(25) $$A_{p}(u,v)=\left\{\begin{array}{ll} 0, & \text{if}\sqrt{(u\Delta u{)}^{2}+(v\Delta v{)}^{2}}>f_{u}\text{or}<f_{l}\\ 1, & \text{otherwise} \end{array}\right.$$

where $\Delta u=\Delta v=\frac{1}{S}$. By multiplying the bandpass filter by the fourier transform of the field in the frequency domain, the frequency components both above the cutoff frequency $f_{u}$ and below the cutoff frequency $f_{l}$ are eliminated (Fig. 14(c) and (g)). The field at the detector (Fig. 14(d) and (h)) is therefore given by:

(26) $$f_{\text{bp}}(x,y)={ℱ}^{-1}\left[F(u,v)A_{p}(u,v)\right]$$

Note that the DC components (incident field $E_{i}$) are intentionally filtered out in both examples in Fig. 14 to better visualize the pattern of the field in the frequency domain. For demonstration purposes, the field is simulated in a limited spatial resolution (R=128×128) to make the concentric bandpass circle visually bigger since the field of view in the frequency domain is proportional to the spatial resolution.

Bandpass filtering can be easily reduced to a 1D case since the far-field Mie scattering is essentially simulated at the Fourier domain. The bandpass filtered 1D simulation can be acquired simply by multiplying the 1D far-field simulation with the corresponding 1D bandpass filter before applying inverse discrete Hankel transform.

**Fig. 15.**
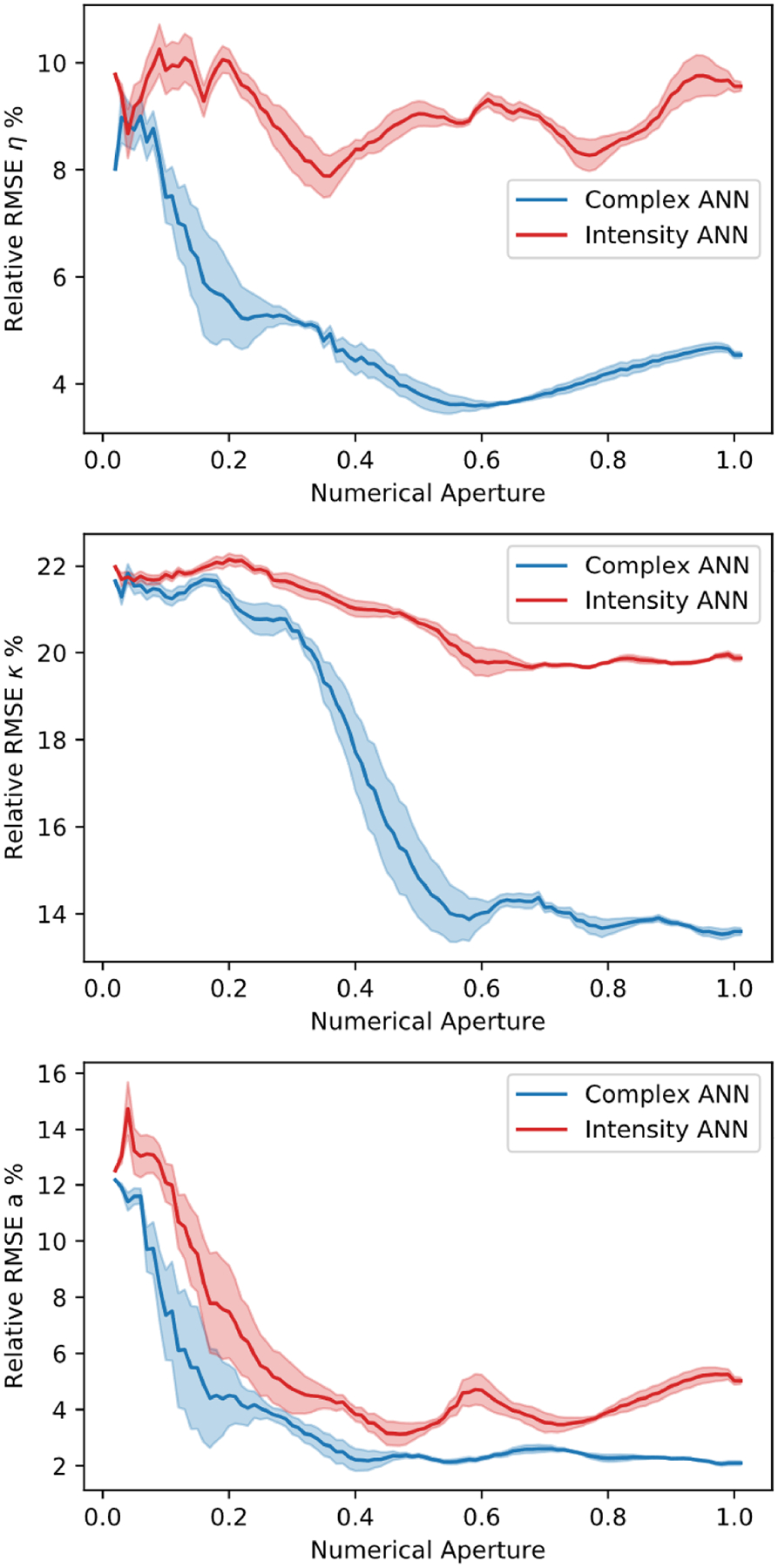
Relative RMSE plots for (top) refractive index m, (middle) extinction coefficient κ, and (bottom) radius a tested on 100 bandpass filtered data sets.

The data generated for bandpass filtering is similar to that of noise test, 100 different NAs ranging in [0.02, 1.01] are added into the training set as the fourth feature. The two ANNs models are then tested with the same number of filters applied to the test set. As shown in Fig. 15, similar to synthetic noisy data, the complex ANN is able to outperform the intensity ANN almost universally.

#### 4) Multi-Sphere Scattering Noise:

**Fig. 16.**
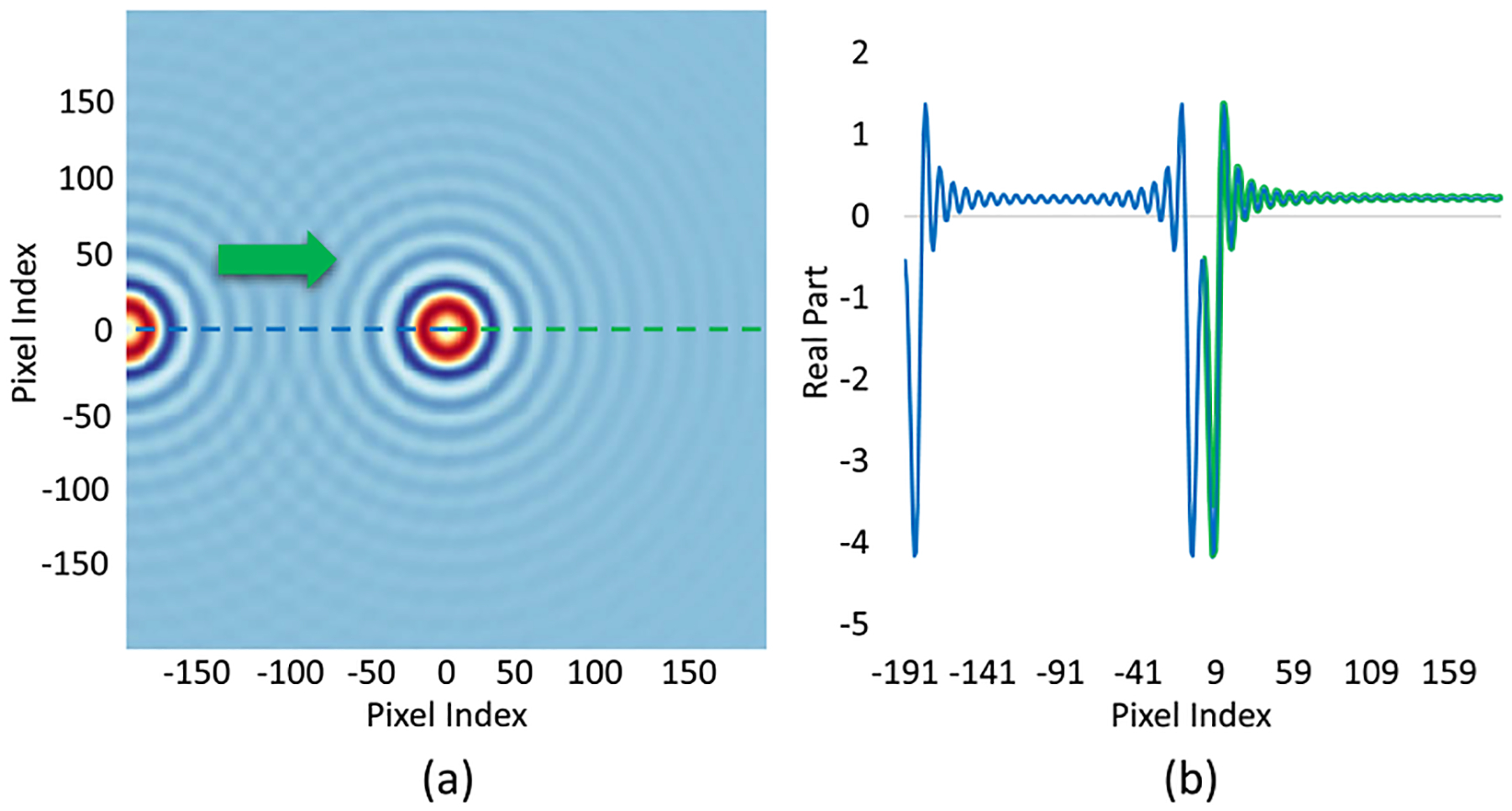
(a) Two spheres with the same material properties are simulated within the same FOV. (b) Corresponding 1D representation of the simulation in (a). Only the right half of the 1D vector is used for training and testing (green part).

Besides randomized synthetic noise and bandpass-filtering, we also tested the model with another form of structural noise by simulating two spheres at once. As shown in Fig. 16(a), two spheres with the same material properties (a=1μm,m=1.5,κ=0.01) are shown in the same FOV. While generating the training set, the sphere placed in the center keeps stationary in all cases. The sphere on the left-hand side moves towards the central sphere for 100 different distances to generate 100 samples with different amount of structural noise. While moving the left sphere closer and closer to the center sphere, more and more structural noise is added onto the field generated by the center sphere (Fig. 16(b)). In this case, only the right half of the 1D representation is used for training and test (green part in Fig. 16(b)) to keep the input data structure consistent.

**Fig. 17.**
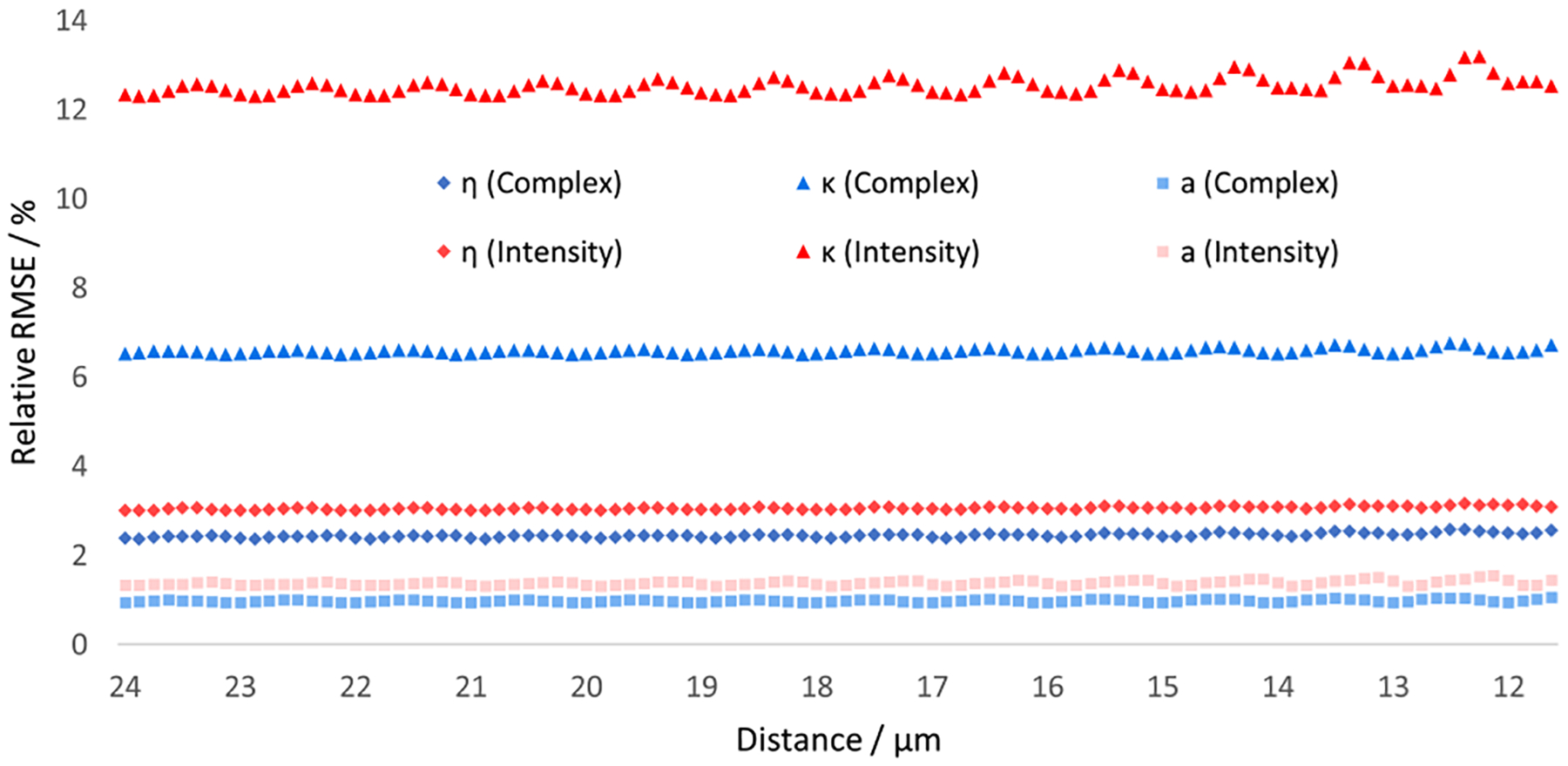
Relative RMSE plot refractive index m, extinction coefficient κ, and radius a tested on 100 different distances between two spheres.

As plotted in Fig. 17, the ANN trained with complex images can outperform ANN trained with intensity images as expected. With a smaller distance between two spheres, the prediction error goes up because more structural noise added to the field generated by the center field, and complex ANN is less sensitive to the noise. In this case, the noise added on to the original signal can be considered as the result of the interference pattern between two spheres. While moving one of the spheres closer to the other, the structural noise is going up and down due to periodic constructive and destructive interference. The error along different distance is hence showing a fluctuating pattern.

## VII. Conclusion

To overcome the limitations in currently available IR imaging systems, we propose a framework for a chemical holographic imaging system (CHIS), which enables phase-sensitive measurements in the Mid-IR region. Scattering models for spherical samples and layered samples are implemented to illustrate and demonstrate the phase-retrieval process of CHIS. The reconstructed complex field provides additional phase information, which opens the door for more innovative and advanced approaches for recovering the sample material properties by extending these principles to infrared spectroscopy.

The far-field simulation for microspheres is implemented to serve as the input for the system when reconstructing spherical samples. The 1D representation of the simulation and the corresponding discrete Hankel transform are extensively studied to simplify the inverse model so that advanced non-linear approaches can be adopted to solve the ill-conditioned inverse problem. The ANN model trained with phase-sensitive measurements can outperform its counterpart trained with traditional intensity representations under all conditions, including applying to synthetic Gaussian-noise data and structured-noise data (bandpass-filtering and multi-sphere interference, for example).

For homogeneous layered samples, on the other hand, by solving an iterative optimization problem, we demonstrate the feasibility and robustness of retrieving both layer-thickness and complex refractive index of a three-layer sample from the recovered phase information, given the effective optical path length of the layers as prior knowledge.

Along with the Mie scattering model and layered model, other types of sample reconstruction can be achieved based on CHIS are not yet being studied, such as multi-layer (more than 3) samples with absorbing materials. There is, therefore, significant room of potential and improvement for optimizing and utilizing CHIS for biomedical imaging.

## Figures and Tables

**TABLE I:** Relative RMSE for Simple ANNs (%)

Material Properties	Complex ANN	Intensity ANN
Refractive Index m	4.69 ± 0.25	6.25 ± 0.18
Extinction Coeff. κ	9.46 ± 0.44	15.90 ± 0.13
Radius a	1.61 ± 0.41	2.77 ± 0.21

**TABLE II:** Relative RMSE for Deep ANNs (%)

Material Properties	Complex ANN	Intensity ANN
Refractive Index m	1.45	3.82
Extinction Coeff. κ	1.12	8.57
Radius a	0.65	2.24
